# Recursive Data Structures in SPARK

**DOI:** 10.1007/978-3-030-53291-8_11

**Published:** 2020-06-16

**Authors:** Claire Dross, Johannes Kanig

**Affiliations:** 8 Microsoft Research Lab, Redmond, WA USA; 9 University of Southern California, Los Angeles, CA USA; AdaCore, 75009 Paris, France

**Keywords:** Deductive verification, Recursive structures, Ownership

## Abstract

SPARK is both a deductive verification tool for the Ada language and the subset of Ada on which it operates. In this paper, we present a recent extension of the SPARK language and toolset to support pointers. This extension is based on an ownership policy inspired by Rust to enforce non-aliasing through a move semantics of assignment. In particular, we consider pointer-based recursive data structures, and discuss how they are supported in SPARK. We explain how iteration over these structures can be handled using a restricted form of aliasing called local borrowing. To avoid introducing a memory model and to stay in the first-order logic background of SPARK, the relation between the iterator and the underlying structure is encoded as a predicate which is maintained throughout the program control flow. Special first-order contracts, called pledges, can be used to describe this relation. Finally, we give examples of programs that can be verified using this framework.

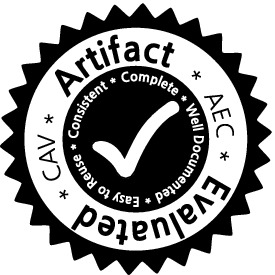



## Introduction

The programming language SPARK [8] has been designed to be amenable to formal verification, and one of the most impactful design choices was the exclusion of aliasing. While this choice vastly simplified the tool design and improved the expected proof performance, it also meant that pointers, as a major source of aliasing, were excluded from the language. While SPARK over the years had seen the addition of many language features, adding pointers just seemed impossible without violating the non-aliasing property. Then came Rust [11] democratizing a type system based on ownership [5]. Taking inspiration from it, it was possible to add pointers to the language in a way that still excludes aliasing. We will give an overview of the rules in this paper.

However, it was unclear if programs traversing recursive data structures such as lists and trees could be supported in this setting. In particular, iteration using a loop requires an alias between the traversed structure and the iterator. In this paper, we detail an approach, inspired by recent work by Astrauskas et al. [1], that enables proofs about recursive pointer-based data structures in SPARK. We have implemented this approach in the industrial formal verification tool SPARK, and, using this tool, developed a number of examples. Some important restrictions remain - we will also discuss them in this paper.

Ada [2] is a general-purpose procedural programming language. The design of the Ada language puts great emphasis on the safety and correctness of the program. This objective is realized by using a readable syntax that uses keywords instead of symbols where reasonable. The type system is strong and strict and many potential violations of type constraints can be detected statically by the compiler. If not, a run-time check is inserted into the program, to guarantee the detection of incorrect situations. 
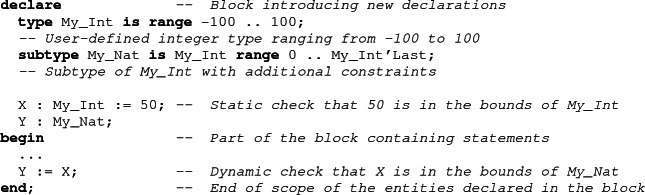
 Ada 2012 introduced contract based programming to Ada. In particular, it is possible to attach pre- and postconditions to subprograms^1^. These conditions can be checked during the execution of the program, just like assertions.

SPARK is the name of a tool that provides formal verification for Ada. It uses the user-provided contracts and attempts to prove that the runtime checks cannot fail and that postconditions are established by the corresponding subprograms. As formal verification for the whole Ada language would be intractable, SPARK is also the name of the subset of the Ada language that is supported by the SPARK tool^2^. This subset contains almost all features of Ada, though sometimes in a restricted form. In particular, expressions should be free from side effects, and aliasing is forbidden (no two variables should share the same memory location or overlap in memory). This restriction greatly simplifies the memory model used in the SPARK tool: any program variables can be reasoned about independently from other variables.

The SPARK tool uses the Why3 platform to generate verification conditions for SMT solvers via a weakest-precondition calculus [4].

## Support for Pointers

Pointers in Ada are called *access types*. It is possible to declare an access type using the access keyword. Objects of an access type are null if no initial values are supplied. It is possible to allocate an object on the heap using the keyword new. An initial value can be supplied for the allocated object. A dereference of a pointer is written as a record component access, but using the keyword all.
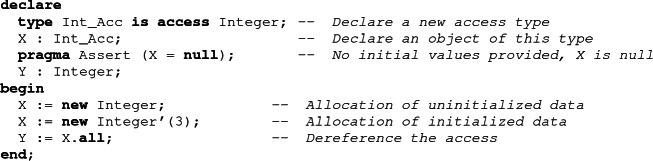
 When a pointer is dereferenced, a runtime check is introduced to make sure that it is not null. Ada does not mandate garbage collection. Memory allocated on the heap can be reclaimed manually by the user using a generic function named Unchecked_Deallocation, which also sets its argument pointer to null. There are several kinds of access types. The basic access types, like Int_Acc defined above, are called pool specific access types. They can only designate objects allocated on the heap. General access types, introduced by the keyword all, can also be used to designate objects allocated on the stack or global data.

Pointers were excluded from the SPARK subset until recently. Indeed, allowing pointers in a straightforward way would break the absence of aliasing in SPARK. In addition, pointers are associated with a list of classes of bugs such as memory leaks, use-after-free and dereferencing a null-pointer.

To support pointers in SPARK, we designed a subset of Ada’s access types which does not introduce aliasing and avoids some pointer-specific issues, while retaining as much expressivity as possible. The first restriction we selected is the exclusion of general access types. This means that SPARK can only create pointers designating memory allocated on the heap, and not on the stack. As a result, pointers can only be made invalid by explicit deallocation, and deallocation of a valid pointer is always legal. To eliminate aliasing between (heap) pointers, ownership rules inspired by Rust have been added on top of Ada’s legality rules. These rules enforce a single writer/multiple readers policy. They ensure that, when a value designated by a pointer is modified, all other objects can be considered to be preserved.

The basis of the ownership policy of SPARK is the move semantics of assignments. When a pointer is assigned to a variable, both the source and the target of the assignment designate the same memory region: assigning an object containing a pointer creates an alias. To alleviate this problem, when an object containing a pointer is assigned, the memory region designated by the pointer is said to be *moved*. The source of the assignment loses the ownership of the designated data while the target of the assignment gains it. The ownership system makes sure that the designated data is not accessed again through the source of the assignment. 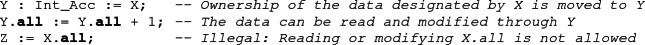 As the ownership policy ensures that no aliasing can occur between access objects, it is possible to reason about the program almost as if the pointer was replaced by the data it points to. When an object containing a pointer is assigned to another variable, it is safe to consider that the designated data is copied by the assignment. Indeed, any effects that could occur because variables are sharing a substructure cannot be observed because of the ownership rules.

Pointers are handled in the verification model of the SPARK proof tool as *maybe*, or *option* types: access objects are either null, or they contain a value. In addition, access objects also contain an address, which can be used to handle comparison (two pointers may not be equal even if the values they designate are equal). When a pointer is dereferenced, a verification condition is generated to make sure that the pointer is not null, so that its value can be accessed. 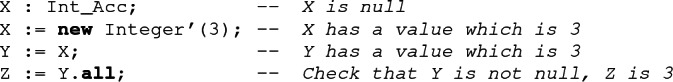 Note that the ownership policy is key for this translation to be correct, as it prevents the program from observing side-effects caused by the modification of a shared reference, which would not be accounted for in the verification model.

## Recursive Data Structures

In Ada, recursivity can only be introduced through pointers. The idea is to first declare a type, but without giving its definition. This declaration, called an *incomplete declaration*, introduces a place-holder for the type, which can only be used in restricted circumstances. In particular, this place-holder can be used to declare an access type designating pointers to values of this type. Using this mechanism, it is possible to declare a recursive data structure, since the access type can be used in the type definition as it comes afterward. 
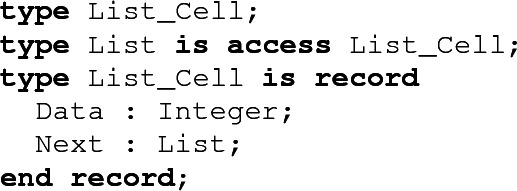
 There are no specific restrictions concerning recursive types in SPARK. However, the ownership policy of SPARK implies that it will not be possible to create a structure which has either cycles (e.g. doubly linked lists) or shared substructures (e.g. DAGs) in it. The ownership policy may also impact how recursive structures can be manipulated. In general, working with such structures involves a traversal, which can be done either recursively, or iteratively using a loop. Algorithms working in a recursive way are generally compliant with the ownership policy of SPARK. Indeed, the recursive calls will allow reading or modifying the structure in depth without having to deconstruct it^3^. 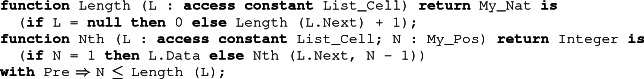 Algorithms involving loops are trickier. The declaration of the iterator used for the loop creates an alias of the traversed data structure. As per SPARK’s ownership policy, this is considered to be a move, so it makes it illegal to access the initial structure. Further assignments to the iterator during the traversal contribute to losing definitively one by one the ownership of every node in the structure, making it impossible to restore the ownership at the end. 
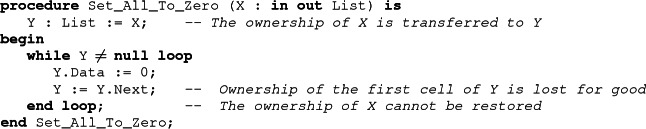
 To traverse recursive data structures, a move is not what we want. Here we need a way to lend the ownership of a memory region for a period of time and automatically restore it at the end. A similar mechanism, called *borrowing*, is available in the Rust language. We have adapted it to SPARK.

## Borrowing Ownership

As Ada is an imperative language, losing the possibility to traverse a linked data structure using a loop was deemed too restrictive. To alleviate this problem, a notion of ownership borrowing was introduced in SPARK. It allows the users to declare a variable, called a borrower, which is initialized with a reference to a part of an existing data structure. To state that this initialization should not be considered a move, an *anonymous access type* is used for the borrower^4^. During the scope of the borrower, the borrowed part of the underlying structure is frozen, meaning that it is illegal to read or modify it. Once the borrower has gone out of scope, the ownership automatically returns to the borrowed object, so that it is again fully accessible. 
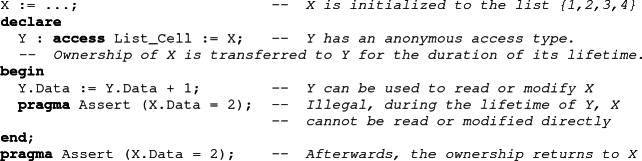
 A borrower can be used to modify the underlying structure. This makes it effectively an alias of the borrowed object. To allow the tool to statically determine the cases of aliasing, SPARK restricts the initial value of a local borrower to be the name of a part of an existing object. This forbids for example borrowing one of two structures depending on a condition.

It is possible to update a borrower to change the part of the object it designates (as opposed to modifying the designated object). This is called a reborrow. In SPARK, the value assigned to the borrower in a reborrow should be rooted at the borrower. This means that reborrows only go deeper into the structure. 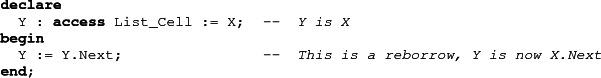 Borrowing can be used to allow simple iterative traversals of a recursive data structure like the loop of Set_All_To_Zero. More complex traversals, involving stacks for example, cannot be written iteratively in SPARK. 
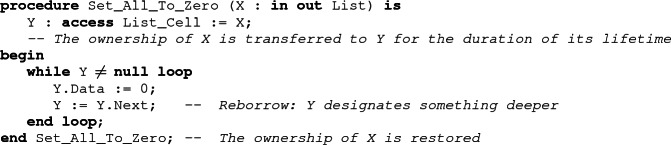
 Using reborrows, local borrowers allow one to indirectly modify a data structure at an arbitrarily-deep position, which may not be statically-known. While in the scope of the borrower, these indirect modifications can be ignored by the analysis, as the ownership policy makes them impossible to observe. However, after the end of the borrow, ownership is transferred back to the borrowed object, and SPARK needs to take into account whatever modifications may have occurred through the borrower. 
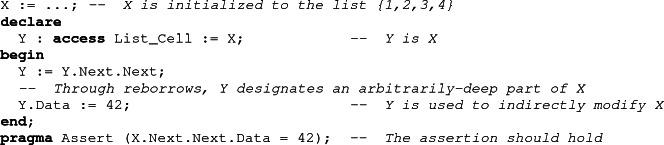
 To be able to reconstruct the borrowed object from the value of the borrower, we must track the relation between them. As this relation cannot be statically determined because of reborrows, SPARK handles it as an additional object in the program. This allows us to take advantage of the normal mechanism for handling value dependent control-flow in SPARK (the weakest-precondition calculus of Why3). The idea is the following. When a borrower is declared in Ada, we create two objects: the borrower itself, which is considered as a stand-alone structure, independent of the borrowed object, and a predicate. The predicate, which we call the borrow relation, encodes the most precise relation between the borrower and the borrowed object which does not depend on the actual value designated by the borrower. The value of the *borrow relation* is computed by the tool from the definition of the borrower, and is updated at each reborrow. Modifications of the underlying data structure don’t impact this relation. At the end of the borrow, the borrowed object is reconstructed using both the borrow relation and the current value of the borrower. 
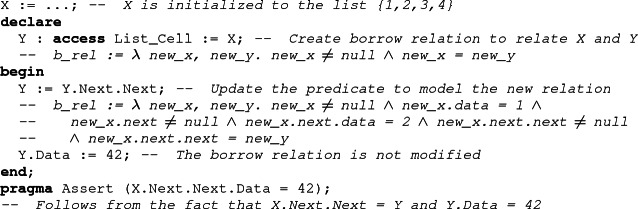


## Describing the Borrow Relation

SPARK performs deductive verification, which relies on user-specified invariants to handle loops. When traversing a linked data structure, the loop body contains a reborrow, which means that the borrow relation is modified in the loop. As a general rule, if a variable is modified in a loop, it should be described in the loop invariant, lest nothing is known about its value afterward. Thus, we need a way to describe the borrow relation in the loop invariant.

As part of their work on the Prusti proof tool for Rust, Astrauskas et al. found the need for a similar annotation that they call *pledges* [1]. In Rust, a pledge is an assertion associated with a borrower which is guaranteed to hold at the time when the borrow expires, no matter what may happen in between. In SPARK, a property guaranteed to hold at the end of the borrow must be a consequence of the borrow relation, since the borrow relation is the most precise relation which does not depend on the actual value of the borrower. Therefore, the user-visible notion of a pledge is suitable to approximate the internally computed borrow relation. Similar to user-provided postconditions, which must be implied by the strongest postcondition computed by a verifying tool, the user-provided pledge should follow from the borrow relation.

Since the Ada syntax has no support for pledges, we have resorted in SPARK to introducing special functions (dedicated to each access type) called pledge functions, which mark expressions which should be considered as pledge expressions by the tool. A pledge function is a *ghost* function (meaning that it is not allowed to have any effect on the output of the program) which has two parameters. The first one is used to identify the borrower on which the pledge should apply, while the second holds the assertion. Note that a call to a pledge function isn’t really a call for the SPARK analyzer. It is simply a marker that the expression in argument is a pledge. 
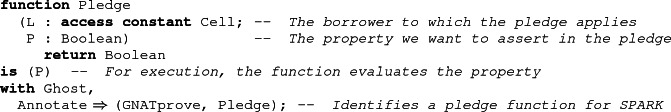
 When a pledge function is called in an assertion, SPARK recognizes it and identifies its parameter as a pledge. It therefore attempts to show that the property is implied by the borrow relation (as opposed to implied by the current value of the borrower). 
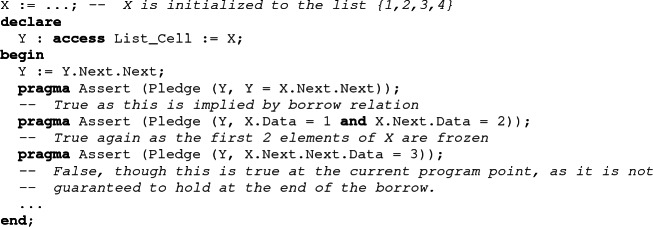
 Using pledges, we can formally verify the Set_All_To_Zero procedure. Its postcondition states that all elements of the list have been set to 0 using the Nth function. To be able to express the loop invariant in a similar way, we have introduced a ghost variable C to count the number of iterations. Its value is maintained by the first loop invariant. The second and third invariants are pledges, describing how the value of X can be reconstructed from the value of the iterator Y. The second invariant gives the length of the list, while the third describes the value of its elements using the Nth function. Elements which have already been processed are frozen by the borrow. Their value is known to be 0. Other elements can be linked to the corresponding position in the iterator Y. 
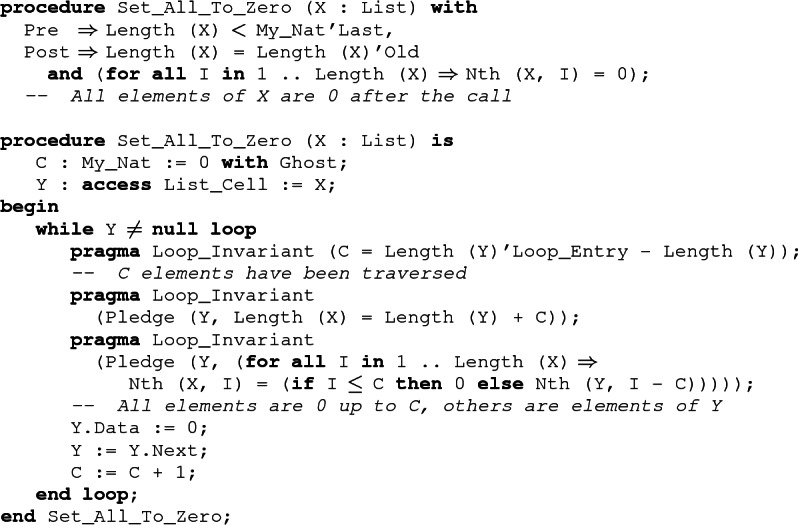
 Note that, in general, it is not necessary to write a pledge to verify a program using a local borrower. Indeed, the analysis tool is able to precisely track the borrow relation through successive reborrows. Pledges need only be provided when the borrow relation itself cannot be tracked by the tool, for example because of a loop, like in our example.

## Evaluation

We could not try the tool on any pre-existing benchmark since SPARK codebases do not have pointers, and Ada codebases usually violate some SPARK rules. In particular, Ada codebases have no reason to abide by the ownership policy of SPARK. So instead, we mostly had to write new tests to assess the correctness and performance of our implementation. The public testsuite of SPARK contains more than 150 tests mentioning access types, be they supported cases or not.

To assess expressivity and provability on programs dealing with recursive data structures, we have written 6 examples, none of them very big, but ranging over various levels of complexity^5^. On all of these examples, we have shown that the runtime checks imposed by the Ada language are guaranteed to pass and that no uninitialized value can be read. In addition, we have manually supplied functional properties.

**Fig. 1.**
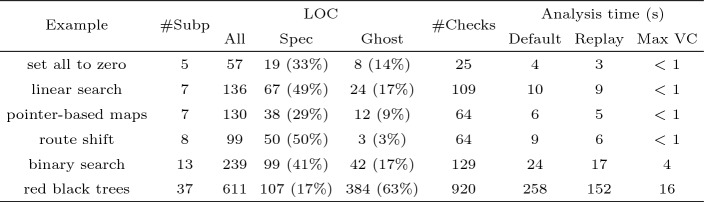
Overview of the examples involving recursive data structures

Figure 1 gives some metrics over these examples. Under the tab Loc are listed the total number of lines of code in the example, the number of lines of specification (including contracts and specification functions), and the number of additional ghost annotations (assertions, loop invariants, ghost variables$\ldots$). The #Checks column gives the number of checks generated by the tool (contracts, assertions, invariants, language defined checks...). In the last three columns, we can see the total running time of SPARK, both from scratch using its default strategy and only replaying the proofs through the replay facility, as well as the maximal time needed to prove a single verification condition.

Though these examples are small, we think they demonstrate that it is possible to define recursive data structures in SPARK, and to verify iterative programs using them. When writing the algorithms, we found that the limitations mostly come from the ownership policy of SPARK. Some data structures are not supported, requiring either to switch to full Ada for their implementations, or to change the algorithm to work around the missing links. In general, we found that the annotation effort required to describe the borrow relations, though non-negligible, was acceptable. In particular, it uses the standard SPARK expressions, with no mentions of memory separation or permission.

## Related Work

Program verification tools for mainstream languages such as C or Java generally support aliasing, because the concept of pointer or reference is more central. They deal with it by modeling the heap. The WP plugin of Frama-C uses by default a *typed memory model* where different arrays are used for the basic types of C [6]. The VerCors [3] toolset handles high-level programming languages, such as Java, by extending the annotation language with separation logic with permission [10]. In SPARK we have chosen a different approach, as we avoid modeling the heap completely by using ownership rules to enforce non-aliasing.

The ownership rules introduced in SPARK are largely inspired by the Rust language [11]. The differences are mostly motivated by the need to comply with the preexisting Ada semantics of pointers. In addition, SPARK was aiming at coming up with a subset as easy to verify as possible. The resulting model is simpler because it does not make lifetime of borrowers explicit, and aliases created through borrows are always statically known.

The Prusti verification tool for Rust [1] allows users to verify that a program complies with its specification. Both tools provide similar guarantees and require similar annotations. However, they differ in their implementation. Indeed, Prusti works by translating separation constraints enforced by the Rust type system to the intermediate verification language of the Viper tool [9]. Our work differs here, as we use the ownership system to abstract away memory related concerns, so that the verification process does not need to be aware of them.

In a recent work [7], Matsushita et al. propose a translation to CHCs for Rust programs. Like in our approach, the restrictions imposed by the ownership policy are key for the soundness of their method. However, while we introduce the notion of borrow relation to be able to use a standard WP calculus, they present a new calculus specifically tailored to Rust references.

## Conclusion

We have presented a recent extension of the SPARK language and toolset to support pointers. It is based on an ownership policy enforcing non-aliasing. To support pointer-based recursive data structures, a restricted form of aliasing is introduced in SPARK through local borrowers, which can be used to iterate through a linked data structure in an imperative way. We have described how local borrowers can be supported by the verification tool, without introducing a memory model, by using a mutable predicate named the borrow relation. This borrow relation can be described when necessary using special annotations named pledges, which solely consist of SPARK standard expressions, and do not expose the underlying verification technique. Our work is available in the 20.1 release of SPARK Pro and will be part of the next community release.

As for future work, we would like to extend the subset of Ada pointers supported in SPARK. In particular, we would like to introduce function pointers to model callbacks, pointers to constants with a more permissive ownership policy, and local borrowing of objects allocated on the stack.

## Footnotes

1 In Ada, a distinction is made between functions that return a value, and procedures, which do not. *Subprogram* is the term that designates both.

2 http://docs.adacore.com/spark2014-docs/html/ug/.

3 In Length and Nth, addition on My_Nat and My_Pos has been redefined to saturate so as to avoid the overflow checking mandated by Ada.

4 A type is said to be anonymous if it does not have a previous declaration. Here access List_Cell is anonymous while List is named.

5 https://github.com/AdaCore/spark2014/tree/master/papers/Pledge2020/examples.
